# Burnout: At times a physical state

**DOI:** 10.1177/10398562251395535

**Published:** 2025-11-12

**Authors:** Gordon Parker, Nicole Russo

**Affiliations:** Discipline of Psychiatry and Mental Health, 7800 University of New South Wales, Sydney, NSW, Australia

**Keywords:** burnout, physical symptoms, self-diagnosis, diagnostic criteria

## Abstract

**Objective:**

While physical symptoms are not uncommonly reported by those experiencing burnout, the syndrome is essentially defined by a set of psychological symptoms. We therefore undertook a study to quantify physical symptoms associated with a burnout syndrome.

**Method:**

A sample of self-diagnosed individuals with burnout were requested to complete data on representative physical symptoms (i.e. headaches, cardiovascular symptoms and falls), illness course variables (i.e. increased rate of infections, being ill more often and taking longer to recover from sickness) as well as being hospitalised as a consequence.

**Results:**

In a refined sample of 317 respondents assigned to a ‘burning out’ stage and 509 to a ‘burnt out phase’, only headaches had a higher prevalence in the first group. Prevalence rates of all symptoms and of compromised health were high while 10% of the combined group reported that their condition had led to them being hospitalised.

**Conclusions:**

Compromised physical functioning would appear to be common in those experiencing a burnout syndrome, and awareness of such a consequence should assist clinical diagnosis of such a condition and might warrant physical symptoms being listed in diagnostic criteria sets as secondary features.

Burnout is generally positioned as a work-related stress response, with ICD-11 positioning it as an ‘occupational phenomenon and with its three symptom domains comprising (i) feelings of energy depletion and exhaustion; (ii) increased mental distance from one’s job or feelings of negativism or cynicism related to one’s job and (iii) reduced professional efficacy’.

Bayes et al.^1^ reviewed studies detailing biological changes reported during burnout states. First, of sustained activation of the autonomic nervous system and dysfunction of the sympathetic adrenal medullary axis leading to alterations in cortisol levels (with representative studies variably reporting increased or decreased levels). Second, of altered immune function and changes in other endocrine systems. The protean consequences – as detailed in the reviewed papers – include increased allostatic load, structural and functional brain changes, systemic inflammation, immunosuppression and increased risks of a metabolic syndrome, type II diabetes, cardiovascular disease and premature death. As any such medical consequences may be rare and, more importantly, slow to manifest, they risk receiving alternate diagnoses (and principally medical ones) rather than being identified as physical concomitants or consequences of burnout.

On some occasions, distinctive physical symptoms can commence abruptly in those experiencing burnout, as now illustrated by two anecdotal reports. In her book, ‘Thrive,’ Arianna Huffington (founder of the Huffington Post) described a rapid onset of her burnout state. Marked by a sudden collapse to the floor of her home office, she hit her head on the corner of her desk and broke her cheekbone.^2^ A second anecdotal example^3^ illustrates the breadth and severity of some physical symptoms. A woman in her forties developed classic burnout symptoms, shortly followed by increasingly frequent and severe respiratory illnesses and intolerance of cold. Over subsequent months, palpitations and episodes of dizziness commenced along with periods of (confirmed) hypoglycemia and severe exertional breathlessness. Taken at a later stage to hospital (when she was unable to get out of bed), her blood pressure was 60/40 when standing, her pulse rate was 170/minute and irregular, while her blood glucose was 2.0 (normal being 4.0 to 7.8 mmol/L). Staff in the intensive care unit was unable to make a finite diagnosis – with one specialist observing that while her symptoms had all the hallmarks of an adrenal crisis, her hormone levels were normal and that he ‘couldn’t figure it out’. Later she had fainting episodes, severe weight loss, periods of nausea and vomiting, while her hair thinned, and she was unable to walk on her own for an extended period.

As a definitive diagnosis of burnout is not always straightforward – to a degree reflecting relatively narrow triadic definitions weighted to psychological symptoms – awareness of physical correlates and their prevalence may assist more accurate identification and diagnosis of the condition, particularly when its presentation is enigmatic.

While physical symptoms have been recognised as a consequence of burnout in some studies,^3–5^ their prevalence in burnout states has not been established and presumably reflects varying definitions of burnout and integral difficulties in differentiating psychological and physical states at times. We therefore undertook a study to determine the prevalence of several physical symptoms informally reported as occurring or as being over-represented in those experiencing burnout and we also included one substantive outcome parameter – hospital admission.

While ‘burnout’ is generally viewed as a singular state, we argue for a two-stage model whereby sufferers experience an initial ‘burning out’ phase, which may progress to a second ‘burnt out’ phase.^3^ Individuals in the initial phase are presumed to be able to revert to their usual functioning if the stressor is removed as their ‘elasticity’ is retained. However, if in a second ‘burnt out’ phase, their elasticity has been lost, and the condition is more finite, less responsive to destressing strategies alone and more likely to require major work–life balance changes. Respecting that model, we evaluated whether the prevalence of physical symptoms differed across such burnout stages.

## Methods

A survey was advertised online via the Black Dog Institute, seeking individuals who self-identified as experiencing burnout and who were over 18 years old and fluent in English. The questionnaire required participants to provide written, informed consent and then respond to a set of questions.

The survey was anonymous and sought demographic and occupational information, the individual’s self-judged phase of burnout (i.e. ‘burning out,’ ‘burnt out’ or ‘not sure’) and nomination of any physical symptoms experienced (from a set list). Listed states were both specific (i.e. headaches, cardiovascular symptoms and infections) and general (i.e. falling ill), and we also inquired about falls, extended recovery time after infections and whether burnout had led to any hospital admission. Study questions were shaped so as to advance participants’ view of symptoms and illness correlates as being directly associated with their burnout state (i.e. ‘Please rate to which degree each of the following features were present in your “burning out” versus “burnt out” stage: (i) never occurred, (ii) occurred only when burning out, (iii) occurred mostly when burning out, (iv) occurred equally in both stages, (v) occurred mostly when burnt out and (vi) occurred only when burnt out)’.

Lastly, participants completed two burnout measures, the Burnout Assessment Tool or BAT^6^ and the Sydney Burnout Measure or SBM,^3^ to judge the likelihood that these self-diagnosed participants had a true burnout syndrome.

## Results

Of the 1351 individuals responding to the online advertisement, 449 (33.2%) did not fully complete the questionnaire and 76 (5.6%) selected ‘not sure’ when asked to nominate their phase of burnout (i.e. burning out or burnt out), leading to such individuals being excluded.

Of the remaining 826 individuals, the 317 who nominated themselves as ‘burning out’ had a mean age of 44.2 (SD 11.1, range 18–65) years, were predominantly female (87.1%) and the mean duration of their burnout was 17.8 (SD 24.9, range = 0.25–180) months. Those 509 who nominated as being ‘burnt out’ had a mean age of 45.0 (SD 11.2, range 18–70) years and were also predominantly female (84.3%). Their ‘burnt out’ phase had a mean duration of 17.9 (SD 29.5, range 0.2–335) months, following a mean burning out phase of 17.9 (SD 29.5, range = 0.2–335) months. When asked if they had had a preceding ‘burning out’ phase, 403 so nominated, 18 (3.5%) denied such a phase and 88 (17.3%) reported as not being sure. To sharpen comparison of the ‘burning out’ and ‘burnt out’ groups, the latter participants were excluded from analysis, resulting in a final sample of 720 participants, with 317 assigned to the ‘burning out’ group and 403 to the ‘burnt out’ group. The ‘burnt out’ group compared to the ‘burning out’ group returned a higher total SBM mean score (i.e. 80.4 vs 66.6) and scored higher on all SBM and BAT measure scales.

The prevalence rates of our evaluated physical symptoms and consequences are reported in Table 1 for both the whole sample and for the ‘burning out’ and ‘burnt out’ sub-samples. The symptom prevalence was distinctive, being (across the whole sample) 89% for headaches, 79% for falling ill more frequently, 78% for taking longer to recover from illnesses, 69% for cardiovascular symptoms, 45% for an increased rate of infections and 28% for falls, while 10% had been hospitalised for their burnout condition. ‘Experiencing headaches’ was the only symptom to have a higher prevalence among those in the ‘burning out’ group while the remaining variables were significantly more prevalent in the ‘burnt out’ group.

**Table 1.** Prevalence of physical symptoms among individuals with burnout in the entire sample and those in separate ‘burning out’ and ‘burnt out’ sub-groups.

Physical symptoms	Entire sample (*n* = 720)	Burning out group (*n* = 317)	Burnt out group (*n* = 403)	Chi-square (burning vs burnt out groups)
I experienced headaches	89.0	90.5	82.9	8.8*
I fell ill more often	78.7	69.1	81.4	14.7**
It took me longer to recover from sickness	78.1	71.3	77.4	3.5
I experienced cardiovascular symptoms (e.g. chest pains, heart rate too fast/slow and low blood pressure)	69.4	64.0	64.5	0.02
I had lots of infections	44.7	34.7	49.1	15.1**
I was falling over/collapsing due to physical exhaustion	27.9	15.1	33.0	30.1**
I was admitted to hospital due to burnout symptoms	9.6	6.0	9.7	3.3

*p < 0.01 and **p < 0.001.

## Discussion

Before considering findings and implications, we note two study limitations. Firstly, sample members were volunteers with self-diagnosed burnout and without that diagnosis confirmed (and alternate diagnoses excluded) following a comprehensive medical and psychiatric interview. In support of most participants truly having a burnout syndrome, we note that their mean score on the SBM was 73.5 (SD: 17.7, range: 15–102) and that 96% exceeded the previously determined SBM cut-off score of 37 for a likely diagnosis of burnout.^7^ We acknowledge, however, that other psychiatric conditions (e.g. depression and anxiety), certain medical conditions (e.g. hypothyroidism and anaemia) and even treatments (e.g. chemotherapy) can generate false-positive assignment on the SBM and all other burnout measures. Secondly, we did not seek data on other physical health conditions when any such pre-existing states may have contributed to symptom prevalence data. However, and as noted earlier, our study questions were shaped so as to advance participants’ view of symptoms and illness correlates as being direct consequences of their burnout state.

Turning to symptoms, headaches were very commonly reported (i.e. by 89% of the sample) and the only symptom more likely to be reported by those in the burning out group. Its early phase expression and high prevalence may suggest its status as a marker of stress onset. In relation to other symptoms, the sample members common reporting of falling ill more often, of taking longer to recover from illness and having an increased rate of infections argues for compromised immunological functioning, with Bayes et al.^1^ interpreting such changes as reflecting sustained hypothalamic–pituitary–adrenal (HPA) axis suppression of the immune system. The cardiovascular symptoms (and potentially of falling over) are likely to reflect persistent activation of the autonomic system. Such activation leads to an increased heart rate, raised blood pressure and disruption of the parasympathetic nervous system and may explain such symptoms being more prevalent for those in a burnt out phase. A rate of 10% of the sample requiring hospital admission argues again for the severity of burnout states in some individuals, but regrettably our study questionnaire did not seek specific details as to why hospitalisation occurred.

We believe that this is the first study to quantify the prevalence of a set of physical symptoms that are informally reported by many with a burnout syndrome. The high prevalence rate of most symptoms evaluated argues for greater recognition of burnout having distinctive physical symptom risks. Future research should extend the list of candidate physical symptoms while consideration should be given as to whether burnout’s definition might be further enhanced by allowing a set of ‘secondary’ physical symptoms or ‘physical specifiers’ to enrich its definition.

## Data Availability

The data associated with this study are available from the corresponding author upon reasonable request.*
